# Lead in the Excretions

**Published:** 1873-12

**Authors:** 


Lead in the Excretions.—M. Mayencon, as the result of a series of investigations, comes to the following conclusions: 1. The salts of lead are not absorbed by the skin. 2. They are absorbed very slowly, and in very small quantities, by the intestines. 3. The absorbed lead seems especially to impregnate the liver and spleen. 4. Sometimes, after long-continued use of these salts, the kidneys and urine contain traces of lead. 5. Its elimination is prompt and complete, and evidently takes place by the liver.

